# Soluble plantain fibre blocks adhesion and M-cell translocation of intestinal pathogens^☆^

**DOI:** 10.1016/j.jnutbio.2012.02.013

**Published:** 2013-01

**Authors:** Carol L. Roberts, Åsa V. Keita, Bryony N. Parsons, Maelle Prorok-Hamon, Paul Knight, Craig Winstanley, Niamh O′Kennedy, Johan D. Söderholm, Jonathan M. Rhodes, Barry J. Campbell

**Affiliations:** a Gastroenterology, Institute of Translational Medicine, University of Liverpool, L69 3GE Liverpool, UK; b Clinical and Experimental Medicine, Division of Surgery, Faculty of Health Sciences, Linköping University, 581 83 Linköping, Sweden; c Institute of Infection and Global Health, University of Liverpool, L69 3GA, UK; d Provexis Plc, c/o Rowett Institute of Nutrition and Health, AB21 9S, Aberdeen, UK

**Keywords:** ANOVA, analysis of variance, CFU, colony forming units, DMEM, Dulbecco′s modified Eagle′s medium, FAE, follicle-associated epithelium, FBS, fetal bovine serum, LB, Luria-Bertani, M-cell, membranous/microfold cell, MOI, multiplicity of infection, NSP, non-starch polysaccharides, PBS, Phosphate-buffered saline, PP, Peyer′s patches, TEER, trans-epithelial electrical resistance, TEM, transmission electron microscopy., Dietary fibre, Diarrhoea, Enteric infections, Peyer′s patches, M (microfold) cell, Mucosal immunology

## Abstract

Dietary fibres may have prebiotic effects mediated by promotion of beneficial bacteria. This study explores the possibility that soluble plant fibre may also improve health by inhibiting epithelial adhesion and translocation by pathogenic bacteria. We have focussed on soluble non-starch polysaccharide (NSP) from plantain bananas (*Musa* spp.) which previous studies showed to be particularly effective at blocking *Escherichia coli* epithelial adherence. In vitro and ex vivo studies assessed the ability of plantain NSP to inhibit epithelial cell adhesion and invasion of various bacterial pathogens, and to inhibit their translocation through microfold (M)-cells and human Peyer′s patches mounted in Ussing chambers. Plantain NSP showed dose-related inhibition of epithelial adhesion and M-cell translocation by a range of pathogens. At 5 mg/ml, a concentration readily achievable in the gut lumen, plantain NSP inhibited adhesion to Caco2 cells by *Salmonella* Typhimurium (85.0±8.2%, *P*<.01), *Shigella sonnei* (46.6±29.3%, *P*<.01)*,* enterotoxigenic *E.coli* (56.1±23.7%, *P*<.05) and *Clostridium difficile* (67.6±12.3%, *P*<.001), but did not inhibit adhesion by enteropathogenic *E.coli*. Plantain NSP also inhibited invasion of Caco2 cells by *S.* Typhimurium (80.2 ± 9.7%) and *Sh. sonnei* (46.7±13.4%); *P*<.01. Plantain NSP, 5 mg/ml, also inhibited translocation of *S.* Typhimurium and *Sh. sonnei* across M-cells by 73.3±5.2% and 46.4±7.7% respectively (*P*<.05). Similarly, *S.* Typhimurium translocation across Peyer′s patches was reduced 65.9±8.1% by plantain NSP (*P*<.01). Soluble plantain fibre can block epithelial adhesion and M-cell translocation of intestinal pathogens. This represents an important novel mechanism by which soluble dietary fibres can promote intestinal health and prevent infective diarrhoea.

## 1 Introduction

It has long been thought that a high intake of dietary fibre promotes intestinal health. Burkitt noted the low rates of bowel cancer and diverticular disease in Africans and thought this might be due to a rapid colonic transit time related to the bulking effects of fibre [1]. Subsequent studies showing that not all fibre sources provided equivalent defence against colon cancer implied more complex protective mechanisms [2] notably fermentation of fibre to generate short chain fatty acids such as butyrate that act as a carbon and energy source for the colonic epithelium [3]. Another mechanism that has attracted attention has been the “prebiotic” effect — an effect that is mediated by promotion of beneficial bacteria [4]. Here we explore an alternative hypothesis - that dietary fibre, particularly soluble fibre, may inhibit adhesion, invasion and translocation of pathogenic bacteria.

Pathogens may induce diarrhoea either as a consequence of invasion and inflammation or by release of toxins. Even for those that release toxins, close proximity to or adhesion to the mucosa is likely greatly to enhance their effect. Anything that prevents their close apposition to the mucosa may therefore have a beneficial or preventative effect. Natural defences will include the mucus layer but, although this is continuous in the healthy colon, it is discontinuous in the small intestine, particularly overlying the Peyer's patches where mucus-secreting goblet cells are relatively sparse [5].

“Membranous” or “microfold” cells (M-cells) are specialized epithelial cells that account for about 5–10% of the dome epithelium that overlies Peyer′spatches in the distal ileum, and lymphoid follicles, their smaller equivalent in the colon [5]. They are the major site of both antigen and microorganism sampling in the gut and also serve as the principal portal of entry for pathogens, such as mycobacteria, *Listeria* spp., *Vibrio cholerae, Salmonella* spp. and *Shigella* spp. These are translocated across M-cells and delivered to the underlying macrophages [6,7]. Previously, we have validated an in vitro derived M-cell model and have shown that translocation of Crohn′s disease mucosal *Escherichia coli* isolates across M-cells is inhibited by soluble plant fibres, particularly plantain (banana) fibre [8]. Furthermore, the effects were verified in ex vivo studies of human follicle-associated epithelium (FAE) taken from resected intestinal tissue of patients undergoing surgery [8], indicating potential for a therapeutic benefit from dietary supplementation with soluble plantain fibre in Crohn′s disease [9,10]. We have now used these models to investigate the potential protective effects of soluble plantain fibre against M-cell translocation by pathogens.

Bacteria that cause toxin-mediated diarrhoea include enterotoxigenic *E. coli* (ETEC), the commonest cause of traveller′s diarrhoea, and *Clostridium difficile*, the major cause of antibiotic-associated diarrhoea. *C. difficile* mediates damage by local release of enterotoxin (*toxin A*) and cytotoxin (*toxin B*) [11]. Close proximity of *C. difficile* to the host epithelium is almost certainly necessary to produce toxic effects [12] and preventing these interactions should therefore be of therapeutic benefit.

Here we show that soluble plantain fibre at concentrations achievable in vivo is able to prevent the adhesion in vitro to intestinal epithelial cells of *Salmonella enterica* serovar Typhimurium, *Shigella sonnei*, ETEC and *C. difficile*. We also show that soluble plantain fibre can inhibit epithelial cell invasion and translocation across M-cells by *S*. Typhimurium and *Sh. sonnei.*

## 2 Materials and methods

### 2.1 Sampling of human Peyer's patches

Tissue specimens from macro- and microscopically normal terminal ileum were obtained from four patients [two women and two men, median age 79.5 (range 52–89) years] who were undergoing right hemicolectomy for colon cancer. All patients had no signs of generalised disease and none had received preoperative chemo- or radiotherapy. The study was approved by the Regional Human Ethics Committee; Linköping, Sweden. All patients had given their informed written consent.

### 2.2 Bacterial strains and growth conditions

*S.* Typhimurium LT2, *Sh. sonnei* and the enteropathogenic *E. coli* (EPEC) strains D55 and E2348/6 were all obtained from stocks held within the Department of Clinical Infection, Microbiology and Immunology, University of Liverpool. ETEC C410 (serotype O160, ST^+^ and LT^+^) was kindly supplied by Dr. Godfrey Smith (Medical Microbiology, Royal Liverpool & Broadgreen University Hospitals NHS Trust, UK). All were cultured on Luria Bertani (LB) agar plates with overnight incubation in air, at 37 °C. *C. difficile* Type 027 (strain 080042), also supplied by Dr. Godfrey Smith, was grown on Fastidious Anaerobe Agar (Lab M, Bury, UK) under anaerobic conditions. *S.* Typhimurium LT2, transformed with a plasmid carrying the enhanced green fluorescent protein gene e*gfp* (pEGFP; BD Biosciences-Clontech, Mountain View, CA, USA), was used in experiments examining bacterial translocation across ex vivo human FAE.

Prior to infection of cultured epithelial cells, bacteria were washed three times, re-suspended in sterile phosphate-buffered saline (PBS) and adjusted to an OD_550nm_ equating to 1×10^9^ CFU/ml.

### 2.3 Soluble plantain fibre

Soluble non-starch polysaccharide (NSP) from plantain, the banana family (*Musa* spp.) member that is usually cooked as a vegetable, was provided by Provexis Plc (Windsor, UK). Soluble NSP was obtained from Green plantain (ripeness stage 1) flour produced in Ecuador from locally grown cultivars *Musa* AAB (Horn) var. Dominico, with a ratio of acidic:neutral polysaccharides of ~ 9:1. The molecular weight distribution of the polysaccharides is between 900 and ~ 5000 kDa [8]. Plantain was selected as it had previously been found to inhibit adhesion of colonic mucosa-associated *E. coli* to intestinal epithelial cells and translocation across M-cells in vitro [8,13]. Concentrations tested were within the range of intraluminal concentrations that would be readily achievable with dietary supplementation, [8].

### 2.4 Epithelial cell culture

The human colorectal adenocarcinoma cell-line Caco2 (#86010202) and the human Burkitt's lymphoma cell-line Raji B (#85011429) were purchased from the European Collection of Animal Cell Culture (Public Health Laboratory Service, Wiltshire, UK). Caco2 Clone 1 cells (Caco2-cl1), kindly provided by Dr. Elisabet Gullberg (University Hospital Linköping, Sweden), were originally obtained from Dr. Maria Rescigno (European Institute of Oncology, Milan, Italy) [14]. Both Caco2 and Caco2-cl1 were grown and maintained in Dulbecco′s modified Eagle′s medium (DMEM) supplemented with 10% v/v fetal bovine serum (FBS), 4 mM l-glutamine, 100 U/ml penicillin and 100 μg/ml streptomycin. The Raji B cell-line was maintained in RPMI-1640 medium supplemented with 10% FBS, 8 mM l-glutamine, 100 U/ml penicillin and 100 μg/ml streptomycin. Raji B cells were seeded at 3x10^5^/ml and every third day cell suspensions were allowed to settle: Two thirds of the media was replaced with fresh culture media. Every ninth day, cells were split 1:3.

All cells were maintained at 37 °C with 5% CO_2_ in a humidified atmosphere. Culture medium and supplements were supplied by Sigma-Aldrich excepting FBS (Invitrogen; Paisley, Scotland).

### 2.5 Adherence to, and invasion of Caco2 monolayers

Bacterial strains were tested for their ability to adhere to, and/or invade, Caco2 cells in the presence of soluble plantain NSP. Cells were maintained in complete Dulbecco′s modified Eagle medium (Sigma) at 37 °C, 5% CO_2_. Cells were initially seeded into 24-well tissue culture plates (Corning/Costar, High Wycombe, UK) at 5×10^4^ cells per well and grown overnight in Dulbecco′s modified Eagle medium (without antibiotics). The monolayers were then washed twice with sterile PBS. Bacteria were grown overnight on agar, washed twice in sterile PBS, before being added to the well. Bacteria were added to each monolayer to a multiplicity of infection (MOI) of 10. For EPEC studies, bacteria were initially added at MOI of 10 but due to low adherence of EPEC to Caco2 cells, studies were repeated at higher MOI of 100 and 500.

After 4 h of infection at 37 °C, cell monolayers were washed 3 times with sterile PBS. To determine bacterial invasion, cells were treated with fresh culture medium containing 100 μg/ml gentamicin to kill extracellular bacteria. After 1 h at 37 °C, the monolayers were again washed 3 times in sterile PBS. Parallel cell monolayers without gentamicin treatment were used to calculate bacterial adhesion. All monolayers were lysed by adding 1% v/v Triton X-100 for 5 min to release internalized bacteria. Tenfold dilutions of the cell lysate were performed, and 50 μl from each was plated onto LB agar plates. Plates were incubated at 37 °C, and colony-forming units were counted after 24 h.

Giemsa microscopy was performed on cells grown on 13 mm glass coverslips in 24 well culture plates. Monolayers were washed with sterile PBS and medium replaced with DMEM without antibiotics, supplemented with 10% FBS and 1% d-mannose. Cells were pre-treated with plantain NSP (10 mg/ml) or saline vehicle for 30 min, and then inoculated with bacteria (grown overnight in static suspension in LB broth containing 1% v/v d-mannose to inhibit type 1 fimbrial adhesins) at MOI 10 for 90 min to 4 h. Cells were washed three times with sterile PBS to remove non-adherent bacteria, fixed with 70% ethanol and stained with 10% Giemsa for 20 min.

### 2.6 Bacterial translocation across M-cells

M-cells were generated on Millicell-PCF 3 μm pore size Transwell filters (Millipore Ltd; Watford, UK) by co-culture of Caco2-cl1 cells (grown on the apical aspect) and Raji B lymphocytes (on the basolateral aspect). Parallel Caco2-cl1 monocultures (without Raji B cells in the basal compartment) were also generated on Transwell inserts and maintained as for M-cells. TEER was measured throughout, using an EVOM epithelial voltohmmeter (World Precision Instruments, Stevenage, UK), to monitor monolayer integrity. Translocation of *S.* Typhimurium and *Sh. sonnei,* coupled with transmission electron microscopy (TEM), was used to confirm successful generation of M-cells in vitro as previously described [8].

For all M-cell and Caco2-cl1 monoculture translocation experiments, DMEM medium was prepared with 10% FBS and 4 mM l-glutamine only (i.e., without anti-microbial agents). For studies examining the effect of soluble dietary fibres, fresh DMEM (0.5 ml) containing plantain NSP (0-50 mg/ml), was applied to the apical aspect of the cells for 30 min at 37 °C. Confluent monolayers were then infected for 4 h, with 1×10^7^ bacteria (MOI of 10) applied to the apical Transwell compartment (filter area 0.6 cm^2^). After infection, the basolateral medium was harvested and bacteria enumerated following overnight culture on LB agar plates incubated at 37 °C in air. Colony forming units (CFU) of viable bacteria were quantified and data expressed as translocated CFU per cm^2^ filter.

### 2.7 Bacterial translocation across isolated human FAE

Bacterial uptake across FAE from macro- and microscopically normal human terminal ileum was performed in Ussing chambers as described previously [8,15]. Briefly, tissue mounted in chambers was pre-incubated for 30 min with 5 mg/ml plantain NSP. Enhanced green fluorescent protein (EGFP)-expressing *S.* Typhimurium LT2 was added to the mucosal compartment (1×10^8^ CFU/ml) and after 2 h infection time the serosal compartment buffer was sampled and measured in a fluorimeter at 488/520 nm (excitation/emission). Numbers of bacteria translocated to the serosal compartment were enumerated relative to an EGFP-expressing *S.* Typhimurium standard curve, with confirmation by CFU counting. Transepithelial potential difference, short-circuit current (Isc) and TEER was monitored throughout. Following Ussing experiments, tissue viability was assessed by adding forskolin (10 μM) to the apical chamber, which raises levels of cyclic AMP with resultant active net ion transport in viable tissue (as assessed by an increase in Isc). This was followed by histological examination of tissue within each chamber as per Refs. [8,15].

### 2.8 Transmission electron microscopy

Following infection, cell monolayers were fixed in 2% v/v glutaraldehyde and 4% w/v paraformaldehyde in sterile PBS. Cells were incubated in 1% w/v osmium tetroxide for 1 h at room temperature, followed by sequential dehydration in ethanol and acetone. Monolayers were removed from the Transwell and cut into 3 mm wide strips (ensuring the cell monolayer was uppermost) and mounted in Araldite resin. Sections (70 nm) were loaded onto copper grids, stained for 5 min each in Reynold's lead citrate and 5% uranyl acetate, washed in distilled water, air-dried and examined using a FEI 120 kV Tecnai G2 Spirit BioTWIN transmission electron microscope (FEI Company; Hillsboro OR, USA).

### 2.9 Statistical methods

*N* numbers indicate the total number of independent experiments performed, where each experiment was performed at least in triplicate for any individual treatment group. For the isolated human tissue experiments, *N* is the number of patients. Independent sample groups were assessed for normality and equality of variances. For multiple treatment groups, Kruskal–Wallis analysis of variance (ANOVA) was employed, followed by post hoc pairwise comparisons of treatment means (StatsDirect v2.6.2; Sale, UK). For the human Peyer′s patch experiments, treatment groups were analysed using a 2-tailed unpaired *t* test. Differences were considered significant when *P*<.05.

## 3 Results

### 3.1 Enteric gut pathogen adhesion to, and invasion of Caco2 cells are inhibited by soluble plantain fibre

**Fig. 1:**
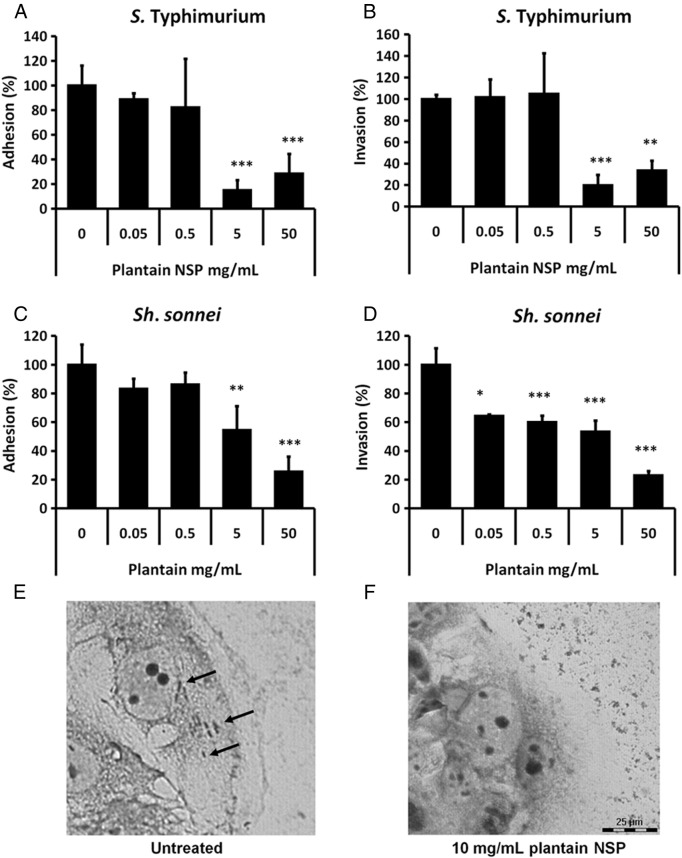
Soluble plantain fibre blocks interaction of *S.* Typhimurium LT2 and *Sh. sonnei* with intestinal epithelial cells in vitro. (A) Adhesion of, and (B) invasion by *S.* Typhimurium LT2 to Caco2 cells is inhibited in the presence of plantain NSP. (C) Adhesion of, and (D) invasion by *Sh. sonnei* to confluent Caco2 cell monolayers is inhibited in the presence of plantain NSP. Adhesion and invasion are both expressed relative to control adhesion in the absence of plantain NSP (set at 100%) (*N*= 3, with minimum *n*= 3 replicates for each treatment group; *P<.05; **P<.01; ***P<.001; ANOVA). (E and F) Giemsa staining of *Sh. sonnei* infected Caco2 cells (70% confluence), in the (E) absence and (F) presence, of 30 min pre-treatment with 10 mg/ml plantain NSP. Solid black arrows indicate intracellular *Sh. sonnei.*

Soluble plantain NSP, at 5 and 50 mg/ml, inhibited *S.* Typhimurium LT2 adhesion and invasion of Caco2 cells (Fig. 1A and B). Similar results were obtained using Caco2-cl1 cells used to generate M-cell cultures (data not shown). Adhesion of *Sh. sonnei* to Caco2 cells was also inhibited by plantain at 5 and 50 mg/ml (Fig. 1C), with *Sh. sonnei* invasion into Caco2 cells significantly inhibited by plantain NSP at concentrations as low as 0.05 mg/ml (Fig. 1D). Blockade of adherence and invasion to Caco2 cells by plantain NSP was confirmed by Giemsa staining and light microscopy (Fig. 1E and F).

**Fig. 2:**
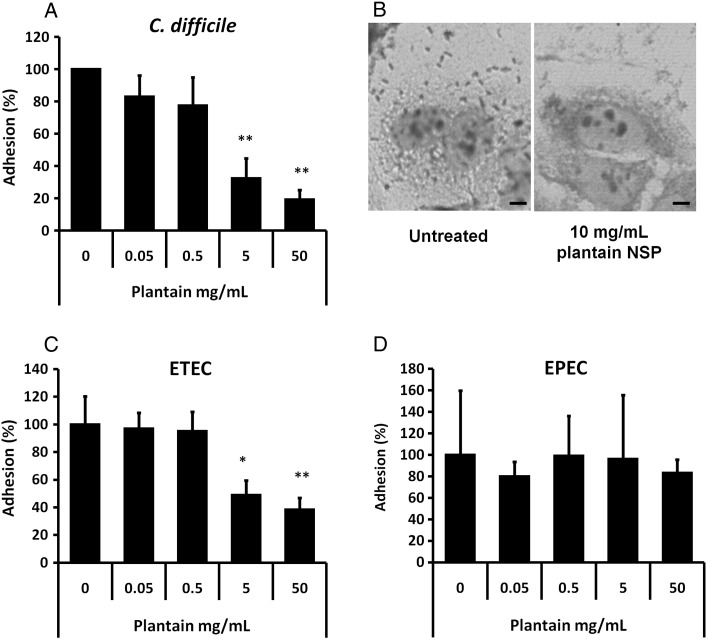
Soluble plantain fibre blocks adherence of *C. difficile* and ETEC but not EPEC. (A) Adhesion of antibiotic-associated diarrhoeal pathogen *C. difficile* to confluent Caco2 cell monolayers was inhibited in the presence of plantain NSP in a dose dependant manner (*N*= 4 separate experiments, with minimum *n*= 3 replicates for each treatment group). (B) Giemsa-stained *C. difficile* infected Caco2 cells (70% confluence) in the absence and presence of 10 mg/ml plantain NSP. Bar=5 μm. (C) Adherence of traveller′s diarrhoea-associated ETEC was also blocked by soluble plantain fibre. (D) Adherence of EPEC to Caco2 cells was not inhibited by soluble plantain fibre (*N*= 3). For all, *P<.05; **P<.01; ***P<.001; ANOVA.

The adhesion of *C. difficile* to Caco2 cells was inhibited 67.6±12.3% and 80.9±5.9% by soluble plantain NSP at 5 and 50 mg/ml respectively (Fig. 2A and B), as was adhesion of the ETEC strain investigated (Fig. 2C). Conversely, soluble plantain NSP had no significant effect upon adhesion to Caco2 cells of EPEC strain D55 (Fig. 2D) nor another EPEC strain tested, E2348/69 (data not shown).

### 3.2 Salmonella and Shigella translocation across M-cells is inhibited by soluble plantain fibre

Both *S.* Typhimurium LT2 and *Sh. sonnei* showed markedly increased translocation through M-cell monolayers (9.63±1.42 and 8.63±1.55 fold respectively) when compared with parent Caco2-cl1 monocultures, indicating successful generation of M-like cells. Interestingly, ETEC were also observed to translocate across M-cells compared to Caco2-cl1 monocultures (3.59±0.37 fold increase (mean±S.E.); *P*<.001 ANOVA).

**Fig. 3:**
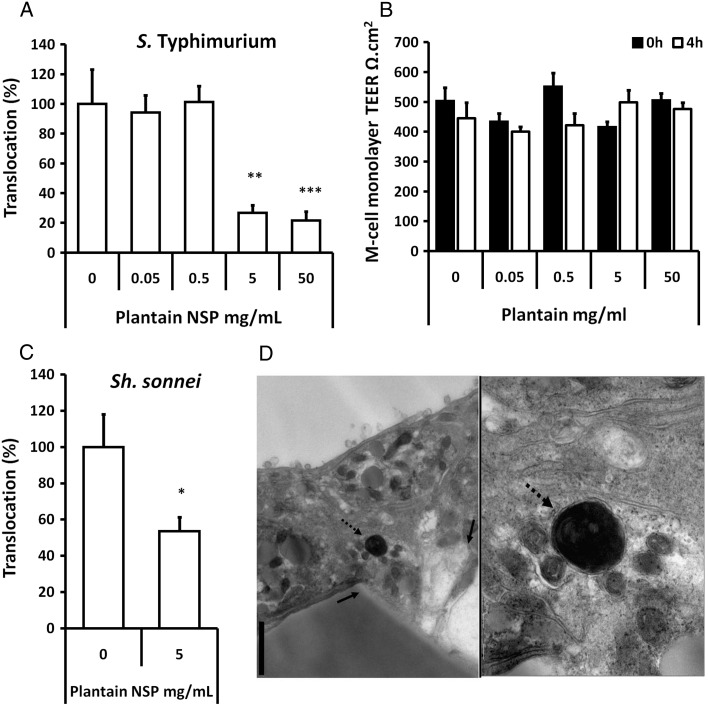
Plantain NSP blocks translocation of enteric gut pathogens across M-cells in vitro. (A) Translocation across M-cells of *S.* Typhimurium is inhibited in the presence of plantain NSP. (B) TEER measurements before (0 h) and after (4 h) infection with *S.* Typhimurium reveal no significant loss of monolayer integrity during infection (*N*= 3), this was also true for M-cell infection with *Sh. sonnei* (data not shown). Translocation is expressed relative to M-cells in the absence of plantain NSP. (*N*= 3, with minimum *n*= 3–5 replicates). (C) Translocation of *Sh. sonnei* across M-cells in vitro was also blocked by plantain NSP. For all, *P<.05; **P<.01; ***P<.001; ANOVA. (D) TEM of a transverse section of M-cells infected with *Sh. sonnei* reveals internalised bacteria (dashed arrow). Solid arrows indicate the aperture of a pore within the Transwell membrane. Bar=1 μm.

Pre-treatment of M-cells for 30 min with soluble plantain fibre, at 5 mg/ml, significantly blocked translocation of *S.* Typhimurium (Fig. 3A); *n*= 3, *P*<.01 ANOVA. At all concentrations tested, soluble plantain NSP had no significant effect upon monolayer TEER (Fig. 3B). Plantain NSP also blocked *Sh. sonnei* translocation across M-cells (Fig. 3C). TEM was used to confirm *Sh. sonnei* within M-cells (Fig. 3D). Similar inhibition of both *S.* Typhimurium and *Sh. sonnei* translocation across parent Caco2-cl1 monocultures (albeit at lower bacterial numbers) was also observed with soluble plantain fibre treatment (data not shown). Plantain NSP also significantly inhibited the translocation of ETEC observed across M-cells at concentrations of 0.5 mg/ml (48.8±15.6% reduction, *P*<.01 ANOVA; *n*= 5) through to 50 mg/ml (18.8±3.5% reduction, *P*<.0001) compared to untreated control (100%).

### 3.3 Salmonella translocation across human Peyer′s patches is inhibited by soluble plantain fibre

**Fig. 4:**
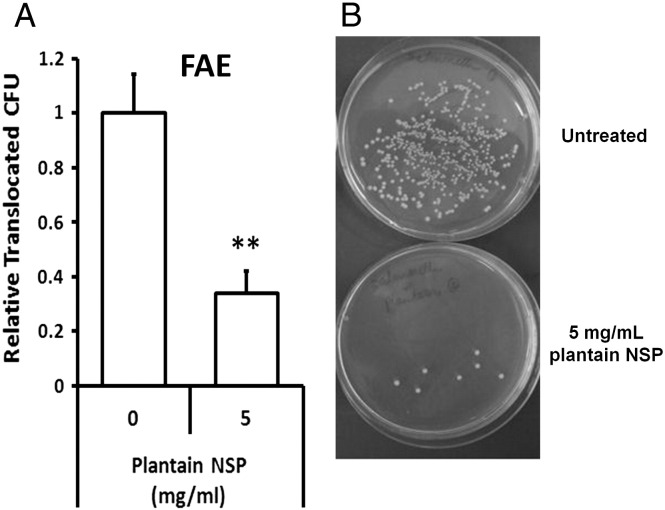
Plantain NSP blocks translocation of *S.* Typhimurium across human Peyer′s patches in Ussing chambers. (A) EGFP-expressing *S.* Typhimurium translocation through follicle-associated epithelium (FAE) of ex vivo human ileal Peyer′s patches (*N*= 4) is inhibited by the presence of 5 mg/ml plantain NSP. **P<.01; 2-tailed unpaired *t* test. (B) Overnight culture of Ussing chamber serosal medium following 2 h translocation of EGFP-expressing *Salmonella* across isolated human FAE, in the absence or presence of soluble plantain fibre.

Translocation of EGFP-expressing *S.* Typhimurium across human ileal FAE was significantly reduced in the presence of 5 mg/ml plantain NSP, to 34.1±8.1% of control levels without plantain treatment (100%; 6.12±0.86×10^5^ bacteria/ml/2 h); *N*= 4, *P*<.01 [2-tailed unpaired t-test] (Fig. 4). TEER was maintained throughout all experiments. Histological assessment verified presence of FAE in each chamber.

## 4 Discussion

Pathogen-related diarrhoea causes about 1.8 million deaths across the world each year [16]. Antibiotics are often used as prophylaxis but increase the likelihood of antibiotic-resistant strains as well as increasing risk of *C. difficile*-associated diarrhoea. The studies presented here suggest that dietary supplementation with soluble plant fibres such as those from plantain bananas may have potential as prophylaxis against intestinal pathogens. Such fibres are capable of passing through the stomach and small intestine without being substantially digested [17]. The fibres may then act as a fermentable substrate for bacteria in the large intestine however, in vitro modelling of soluble plantain NSP breakdown by mixed faecal bacteria obtained from healthy volunteers has shown that 25–75% of ingested plantain NSP is likely to avoid fermentation in the human colon [8,18]. In vitro studies suggest that Bacteroides are the major fermenters of plantain NSP, whereas members of other key groups such as Ruminococci (including *Roseburia* spp. or *Faecalibacterium prausnitzii*) and Bifidobacteria, both targets for prebiotic supplementation, are unable to utilise plantain NSP [18]. Whilst previous investigations have often focussed on the beneficial effects of NSP mediated either by encouraging the growth of probiotic bacteria, i.e., prebiotic effects, or by generation of short-chain fatty acids such as butyrate, the data presented here suggest another potentially beneficial mechanism of soluble NSP: the prevention of bacterial adherence to the gut epithelium.

Soluble plantain fibre at concentrations of 5 mg/ml or higher is shown here to inhibit the adhesion to Caco2 cells of diarrhoeal pathogens including *S.* Typhimurium, *Sh. sonnei*, ETEC and *C. difficile.* This concentration of fibre should be readily achievable by dietary supplementation, e.g. 5 g twice daily, even in the distal colon after partial fermentation. Assuming passage of one litre of fluid daily into the caecum, intake of 5 g plantain NSP twice daily, with 25% fermentation, would produce NSP concentrations of 10 and 7.5 mg/ml in the caecum and rectum, respectively [8].

ETEC and *C. difficile* elicit their pathogenic effects via release of toxins, however both pathogens also adhere to the epithelium and blockade of this adherence is likely to substantially reduce local concentrations of their toxins [19].

Invasion into Caco2 cells of *S.* Typhimurium and *Sh. sonnei* was also inhibited by the presence of soluble plantain fibre at 5 mg/ml. At this concentration, soluble plantain fibre also blocked translocation across M-cells by *S.* Typhimurium and *Sh. sonnei* and studies using ex vivo human ileal Peyer′s patches mounted in Ussing chambers confirmed blockade of translocation of *S.* Typhimurium across the follicle-associated epithelium (FAE).

The juice from boiled green bananas, that would contain soluble fibre, has previously been reported to reduce the severity and duration of persistent diarrhoeas [20–22] including shigellosis [23]. Our own studies have shown that soluble dietary fibre from other plant sources such as broccoli may also block bacteria-epithelial adherence although other plant fibres, including leek and apple, did not have this effect [8].

Soluble plantain NSP is here shown to block adherence of ETEC but not of EPEC to Caco2 cells. Localised adherence of EPEC strain E2348/69 to Caco2 cells is known to be dependent on type IV bundle-forming pili (BFP), with α1-bundlin, a major protein subunit of BFP, being the key adhesin mediating early localised adherence to Caco2 and HEp-2 intestinal cell-lines [24,25]. Alpha bundlins are blocked specifically by *N*-acetyllactosamine (LacNAc) and likewise other adhesins of EPEC, such as intimin, are also inhibited by LacNAc [25]. It seems that this very specific adhesion mechanism is not inhibited by any of the polysaccharides present within soluble plantain fibre.

There is a growing interest in the possible role of epithelial-associated bacteria in the pathogenesis of colorectal cancer [26,27]. They may for example interact with Toll-like receptors with consequent signalling via the MyD88 pathway and epithelial NFκB activation, key steps in experimental cancer pathogenesis [28–31]. It seems highly plausible that the variable protective effect of dietary fibres seen in epidemiological studies could be mediated at least in part by their ability to block epithelial recruitment of bacteria and this is likely to vary considerably between different fibre types. In addition, soluble fibre fermented to fatty acids in the human colon by the resident microbiota would also likely benefit colonocyte health [32]. Further investigation of this might help to clarify the evidence base for dietary associations with colorectal cancer.

Clinical trials are now indicated to assess the efficacy of soluble plantain fibre and other soluble plant fibres for their ability to prevent pathogen-related illness by blocking adhesion and/or invasion. We have suggested that such an effect of soluble plant fibre might be termed “contrabiotic” [33]. Antibiotic-associated diarrhoea and traveller′s diarrhoea would be obvious targets.
